# Genetic overlap and shared risk loci between autism spectrum disorder and cardiometabolic traits

**DOI:** 10.1038/s41380-026-03563-x

**Published:** 2026-04-20

**Authors:** Gerard Muntané, Alexey Shadrin, M. Guardiola-Ripoll, Kevin S. O’Connell, Oleksandr Frei, Terje Naerland, Elisabet Vilella, Ole A. Andreassen

**Affiliations:** 1 Hospital Universitari Institut Pere Mata (HUIPM), Institut de Recerca Biomèdica Catalunya Sud (IRBCatSud), Reus, Spain; 2 Departament de Medicina i Cirurgia, Universitat Rovira i Virgili, Catalonia, Spain; 3 Centro de Investigación Biomédica en Red de Salud Mental (CIBERSAM), Instituto de Salud Carlos III, Madrid, Spain; 4 Institut de Biologia Evolutiva (UPF-CSIC), Department of Medicine and Life Sciences, Universitat Pompeu Fabra, Parc de Recerca Biomèdica de Barcelona, Barcelona, Catalonia Spain; 5 K.G. Jebsen Centre for Neurodevelopmental Disorders, Institute of Clinical Medicine, University of Oslo, Oslo, Norway; 6 Centre for Precision Psychiatry, Division of Mental Health and Addiction, Oslo University Hospital and Institute of Clinical Medicine, University of Oslo, Oslo, Norway; 7 Department of Pharmacy, Section for Pharmacology and Pharmaceutical Biosciences, University of Oslo, Oslo, Norway; 8 NevSom Department of Rare Disorders, Oslo University Hospital, Oslo, Norway

**Keywords:** Autism spectrum disorders, Genetics

## Abstract

Autism spectrum disorder (ASD) is a neurodevelopmental condition affecting 2% of the global population. Beyond core symptoms such as social communication deficits and repetitive behaviors, individuals with ASD are at increased risk of cardiometabolic comorbidities, including obesity, diabetes, and cardiovascular disease. Here, we investigate the shared genetic architecture between ASD and cardiometabolic traits using large genome-wide association studies datasets and advanced statistical approaches: the bivariate causal mixture (MiXeR) model and pleiotropy-informed conditional false discovery rate (pleioFDR). Our results show significant polygenic overlap between ASD and several cardiometabolic phenotypes, despite almost negligible genetic correlation between the traits. Specifically, we observed positive genetic correlations within the shared component for ASD and metabolic traits, such as body mass index (rg=0.03), type 2 diabetes (rg=0.23), and total cholesterol (rg=0.78). In contrast, negative correlations emerged between ASD and cardiovascular traits, including diastolic and systolic blood pressure (rg = -0,22, for both), pulse pressure (rg = -0.25), and coronary artery disease (rg = -0.90). Finally, we identified 100 shared loci between ASD and cardiometabolic traits, mapping to 124 genes and suggesting shared biological mechanisms underlying these phenotypes and pointing to potential therapeutic targets. Shared loci between ASD and metabolic traits predominantly showed concordant effects, whereas those overlapping with cardiovascular traits—particularly blood pressure–related traits—tended to exhibit discordant effects. Together, these findings deepen our understanding of the biological connections between ASD and cardiometabolic comorbidities and may help inform more personalized strategies for managing ASD and its associated long-term health risks.

## Introduction

Autism Spectrum Disorder (ASD) is a complex neurodevelopmental condition that affects approximately 2% of the global population [1]. While primarily characterized by challenges in social communication and repetitive behaviors, ASD is often associated with various medical, neurological, and psychiatric comorbidities that impact the quality of life and longevity of affected individuals [2–4]. The etiology of ASD is multifaceted and involves complex interactions between genetic and environmental factors. With an estimated heritability of approximately 80% [5, 6], recent genome-wide association studies (GWAS) and sequencing efforts have revealed the mixed genetic nature of ASD, with both rare and common genetic variants contributing to risk [7–11].

Emerging evidence suggests that individuals with ASD have a significantly higher prevalence of various physical health comorbidities, which affect multiple systems and represent an important health burden [12]. In particular, individuals with ASD are at an elevated risk for cardiometabolic comorbidities, with studies reporting higher incidences of metabolic syndrome and other conditions such as obesity, type 2 diabetes mellitus (T2D), hypercholesterolemia, dyslipidemia (with risk increases of between 1.41 and 2.7-fold), as well cardiovascular traits, such as hypertension and cardiovascular disease (with incremental risks of 2.2-fold) in ASD populations compared to neurotypical peers [13–19]. While cardiovascular disease is traditionally classified as a cardiovascular trait due to its direct impact on the heart and blood vessels, it is closely linked to metabolic risk factors, which contribute to the progression of atherosclerosis [20, 21]. In contrast, blood pressure-related traits are more influenced by vascular resistance, kidney function, and autonomic regulation, highlighting distinct biological pathways [22]. This heightened risk appears to be multifactorial, with contributions from lifestyle factors such as diet and physical activity [16, 23], medication use [15, 24], and genetic predisposition. Specific loci, such as the 16p11.2 deletion and duplication, have been associated with obesity and other cardiometabolic conditions in ASD [25, 26]. Interestingly, other neuropsychiatric conditions, such as schizophrenia, also exhibit substantial genetic overlap with cardiometabolic traits, despite low genetic correlation (e.g., ranging from approximately 0.40 for total cholesterol to –0.17 for BMI) [27]. However, the extent to which genetic factors contribute to the co-occurrence of ASD and cardiometabolic diseases remains underexplored [28].

In the current study, we leverage large-scale GWAS datasets to explore the common genetic architecture and shared genetic variants between ASD and various cardiometabolic traits, including metabolic and cardiovascular phenotypes. By elucidating the complex relationship between these conditions, our research aims to identify the molecular mechanisms linking ASD and cardiometabolic health to gain a more comprehensive understanding of their relationship and contribute towards the identification of potential new targets for intervention.

## Methods

### GWAS samples

We utilized summary statistics from GWAS of independent samples of ASD and multiple cardiometabolic phenotypes. The ASD GWAS data were obtained from Grove et al. 2019 [9], comprising 18,382 individuals with ASD and 27,969 controls, all of European ancestry. The phenotypes analyzed were extracted from large-scale GWAS datasets, which were entirely of samples of European ancestry, or, in the case of multi-ancestry GWAS, the analyses were conducted in the European subsets (Table S1). The included metabolic phenotypes were body mass index (BMI; 795,640 individuals) [29], total cholesterol (TC), high-density lipoprotein (HDL) (European ancestry subset: ~216,000 individuals) [30], T2D (European ancestry subset: 80,154 cases; 853,816 controls) [31]. The cardiovascular traits were pulse pressure (PP; 745,820 individuals), systolic and diastolic blood pressure (SBP; 745,820, and DBP; 757,601 individuals) [32], and coronary artery disease (CAD; 71,602 cases; 260,875 controls) [33]. All GWASs included rigorous quality control procedures and accounted for population by adjusting for genetic principal components. Based on the description of the samples in the original studies, no sample overlap was detected between the ASD GWAS and any of the cardiometabolic GWASs.

### Statistical analyses

To assess polygenic overlap between ASD and cardiometabolic phenotypes, we used bivariate causal mixture (MiXeR) model [34, 35]. It utilizes summary statistic data from two GWAS to estimate the number of phenotype-influencing genetic variants (polygenicity) and the variance of their effect sizes (discoverability) for each of the phenotypes, as well as to quantify the proportion of variants shared between the two phenotypes. In the context of the MiXeR method, phenotype-influencing variants (also referred to as “causal” or non-null variants) are those with non-zero direct effects (not induced by linkage disequilibrium) on the phenotype. The model is not restricted to locus lead variants or variants exceeding a certain significance threshold. Variant effect sizes are modeled using point-normal mixture priors, allowing variants shared between phenotypes to have both concordant and discordant directions of effect while accounting for linkage disequilibrium (LD) and sample overlap between the analyzed GWAS. LD structure was estimated using unrelated individuals of European ancestry from the 1000 Genomes Project Phase 3 reference panel. More details on the method are available in a dedicated review paper [35]. Univariate MiXeR analyses were performed for each phenotype to estimate the SNP-based heritability (h^2^SNP) and polygenicity, providing insight into how genetically complex a trait is. Polygenicity is reported as the number of strongest phenotype-influencing variants required to explain 90% of the phenotype’s SNP-based heritability. A threshold of 90% is used to prevent extrapolating model parameters to variants with vanishingly small effects [35]. The ability of the MiXeR model to predict the actual GWAS data, that is model fit, was assessed using log-likelihood plots and Akaike Information Criterion (AIC). When evaluating the model we followed guidelines as described in Van Der Meer et al. [35].

Genome-wide genetic correlations (rg) across all SNPs were estimated using both MiXeR and LD score regression [36], and correlations of effect sizes within the common genetic component were assessed using MiXeR. While the genetic overlap focuses on the regions influencing various traits and estimates the number of variants shared, it does not inform on the direction and consistency of the effects across the genome [37]. LD structure was estimated using unrelated subjects of European ancestry from the 1000 Genomes Phase 3 genotype reference panel. Major histocompatibility complex region (MHC, chr6: 26,000,000-34,000,000, GRCh37 genomic build) was excluded from all GWAS summary statistics because of its intricate poorly modeled LD structure. Finally, visual representations of the results were generated using Venn diagrams.

The conjunction FDR analysis as implemented in pleiotropy-informed conditional false discovery rate (pleioFDR) software [38] was used to identify pairwise shared genetic loci between ASD and each of the secondary cardiometabolic traits evaluated (at conjFDR < 0.05), even if they do not reach genome-wide significance in either GWAS individually [39]. Genomic regions with strong associations with MHC (chr6: 25,119,106–33,854,733, GRCh37) and 8p23.1 (chr8: 7,200,000–12,500,000, GRCh37) were excluded before fitting the null model to avoid inflated FDR estimates. Conditional Q-Q plots were used to visualize cross-trait enrichment, showing an increase in SNPs associated with one phenotype as the strength of association with a secondary phenotype increased. Independent genomic loci and corresponding lead and candidate variants were defined using FUMA [40]. The directional effects of the shared loci were then assessed by comparing the effect direction of corresponding lead variants in the original GWAS summary statistics. conjFDR analysis requires non-overlapping samples, otherwise results might be inflated.

### Functional analyses

To explore the biological mechanisms associated with the identified genetic loci, we performed an enrichment analysis of Gene Ontology (GO) functions and pathways using FUMA [40]. This analysis focused on genome-wide cross-phenotype significant pleiotropic SNPs, and incorporated various functional annotations, including regulatory functions, chromatin states and Combined Annotation Dependent Depletion (CADD) scores [41]. CADD scores are a widely used metric that integrates multiple sources of genomic information to estimate the deleteriousness of genetic variants. We prioritized SNPs with CADD scores > 12.37 as indication of their likely deleterious effects [42]. Candidate SNPs were mapped to putative causal genes through positional mapping, and both nearest genes and functional consequence of each SNP on gene functions were annotated based on FUMA. We used the full set of protein-coding genes as background for enrichment analyses.

To further explore the translational implications, we used the Genome for Repositioning Drugs (GREP) framework (https://github.com/saorisakaue/GREP) to identify drugs that target genes shared by ASD and cardiometabolic phenotypes. We evaluated enrichment of drug-target gene sets across clinical indication categories, defined by the Anatomical Therapeutic Chemical (ATC) classification or International Classification of Diseases, Tenth Revision (ICD-10) codes, using Fisher’s Exact Test. Additionally, we assessed drug-gene interactions (DGIs) for the shared genes using the Drug-Gene Interaction Database (DGIdb; www.dgidb.org). We considered DGIs with an interaction score greater than zero to be meaningful and included only drugs present in the GREP library to ensure consistency across analyses.

## Results

### Genetic overlap between ASD and cardiometabolic phenotypes

The univariate MiXeR results revealed substantial variation in the number of variants accounting for 90% of h^2^SNP across the evaluated phenotypes. ASD and BMI exhibited the highest estimated polygenicity, with 12,200 and 11,000 variants, respectively, contributing to their genetic architecture. In contrast, the other metabolic and cardiovascular traits displayed comparatively lower polygenicity. Blood pressure measures, including PP, SBP, and DBP, showed moderate polygenicity, with 3600, 4400, and 4000 variants, respectively. T2D followed a similar pattern with 2600 variants. CAD involved 1400 variants, while lipid traits, TC and HDL, had the lowest polygenicity, with 200 to 400 variants respectively. These values are summarized in Table S2. Diagnostic univariate Q-Q plots comparing observed versus predicted GWAS p-values are shown in Figure S1. AIC values were consistently positive for all traits, indicating robust model fit for our analyses (Table S2).

**Fig. 1:**
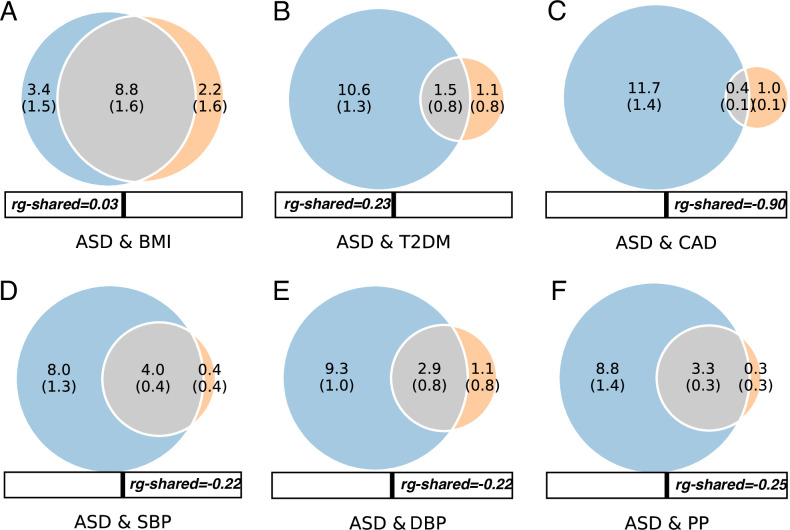
Venn diagrams depicting the shared and unique genetic variants influencing ASD (blue) and various cardiometabolic phenotypes (orange). The diagrams illustrate the polygenic overlap (grey) between ASD and metabolic phenotypes: **A** Body Mass Index (BMI), **B** Type 2 Diabetes Mellitus (T2D); and between ASD and cardiovascular phenotypes **C** Coronary Artery Disease (CAD), **D** Systolic Blood Pressure (SBP), **E** Diastolic Blood Pressure (DBP), and **F** Pulse Pressure (PP). Numbers within each Venn diagram represent the estimated quantity of shared and unique variants (in thousands) contributing to 90% of single-nucleotide polymorphism (SNP)-based heritability for each trait, with the standard deviation across 20 independent MiXeR runs shown in parentheses. Circle sizes reflect the degree of polygenicity for each phenotype. Additionally, estimates of correlation within the shared component between ASD and each cardiometabolic trait (rg-shared) are provided for each trait pair. TC and HDL plots were omitted because they were not reliable (based on log-likelihood plots).

The pairwise total genetic correlations between ASD and the cardiometabolic phenotypes obtained using LD score regression were insignificant for most of the traits, except for significant negative correlations between PP and SBP (P = 9.7e-03 and P = 0.04, respectively; Table 1 and Table S3) that did not survive FDR correction. The genetic correlations within the shared component (represented by the grey areas in the Venn diagrams from Fig. 1) are summarized in Table 2. MiXeR estimated positive genetic correlations within the shared component for ASD and the majority of the metabolic traits: BMI (rg = 0.03), T2D (rg = 0.23), and TC (rg = 0.78), except for a negative genetic correlation with HDL (rg = -0.74, Table 2). In contrast, we observed negative genetic correlations within the shared component between ASD and cardiovascular traits: DBP (rg = -0.22), SBP (rg = -0.21), PP (rg = -0.25), and CAD (rg = -0.9, Table 2). The MiXeR bivariate analyses revealed substantial genetic overlap between ASD and certain cardiometabolic phenotypes (Fig. 1 and Table S4). Notably, 8759 ± 1586 of the 12,200 ASD-influencing variants also influenced BMI (Fig. 1A), representing 72% of the ASD-influencing variants overlapping with BMI. Fewer variants were shared between ASD and other traits: SBP (N = 4045 ± 380), PP (N = 3334 ± 317), DBP (N = 2862 ± 780), T2D (N = 1459 ± 803), and very few variants with TC (N = 147 ± 42), HDL (N = 265 ± 69), and CAD (N = 428 ± 96) (Fig. 1C–F, Table 2). Despite the reduced number of shared variants between ASD and these traits, the proportion of overlapping variants relative to the total polygenicity of these traits was substantial. Nevertheless, the lower polygenicity observed in these cardiometabolic phenotypes, in comparison to BMI, partly explains the reduced absolute overlap (Table 2). Bivariate MiXeR adequately modeled the data for most traits, as indicated by Q-Q plots, log-likelihood curves, and AIC values. The bivariate AIC values (*best_vs_min* and *best_vs_max*) for all traits were positive, indicating an important genetic overlap (supported by *best_vs_min AIC* > 0), but also there were phenotype-specific variants in all analyzed phenotypes (supported by *best_vs_max AIC* > 0, Table S4). However, MiXeR was not able to model the metabolic traits TC and HDL adequately, as suggested by the erratic behavior of the log-likelihood curve shown in Figure S2.

**Table 1:** Independent genetic loci shared between ASD and cardiometabolic phenotypes.

				Genetic correlation (LDSC)		
	Associated phenotype	Shared Loci^a^	Concordant effect (%)	rg	P	FDR P-values
metabolic	BMI	21	48	0.03	0.22	0.41
	T2D	8	50	0.04	0.31	0.41
	TC	3	66	0.02	0.55	0.63
	HDL	5	60	-0.01	0.73	0.73
cardiovascular	DBP	23	48	-0.04	0.26	0.41
	SBP	25	36	-0.07	0.04	0.16
	PP	14	7	-0.08	9.7e-03	0.08
	CAD	1	0	-0.05	0.19	0.41

^a^Independent genomic regions jointly associated with ASD and each cardiometabolic phenotype.

**Table 2:** MiXeR estimates for ASD and cardiometabolic phenotypes.

	Associated Phenotype	Estimated N of variants shared with ASD (SD)	ASD variants shared with cardiometabolic traits (%)	Cardiometabolic variants shared with ASD (%)	Genetic Correlation of Shared Variants (SD)
metabolic	BMI	8759 (1586)	72.5	80	0.03 (0.02)
	T2D	1459 (803)	12	56.7	0.23 (0.21)
cardiovascular	DBP	2862 (780)	23.7	72	-0.22 (0.07)
	SBP	4045 (380)	33.5	91.7	-0.22 (0.02)
	PP	3334 (317)	27.6	91.4	-0.25 (0.04)
	CAD	428 (96)	3.5	31.1	-0.90 (0.11)

### Shared loci between ASD and cardiometabolic phenotypes

In total, we identified 7624 SNPs associated with both ASD and cardiometabolic phenotypes at a conjFDR threshold of <0.05, among which 2754 were unique SNPs. Specifically, for metabolic phenotypes, we identified 363 SNPs shared between ASD and BMI, 334 SNPs with T2D, 18 with TC, and 16 with HDL. Regarding cardiovascular phenotypes, 2346 SNPs were shared between ASD and DBP; 2433 SNPs with SBP; 2112 with PP; and finally, 2 with CAD.

**Fig. 2:**
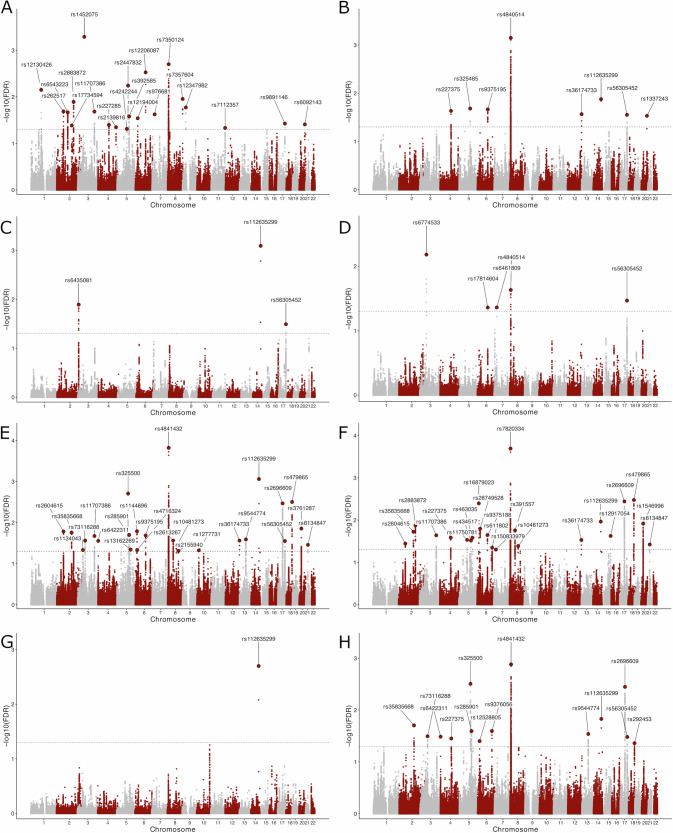
Manhattan plots of common genetic variants jointly associated with ASD and metabolic phenotypes. **A**) Body Mass Index (BMI), **B**) Type 2 Diabetes Mellitus (T2D), **C**) Total Cholesterol (TC), and **D**) High-density lipoprotein cholesterol (HDL); and those jointly associated with ASD and cardiovascular phenotypes: **E**) Systolic Blood Pressure (SBP), **F**) Diastolic Blood Pressure (DBP), **G**) Coronary Artery Disease (CAD), and **H**) Pulse Pressure (PP). Each panel displays the –log_10_ transformed conjFDR values for each single-nucleotide polymorphism (SNP) on the y-axis, with chromosomal positions along the x-axis. SNPs with conjFDR < 0.05 (−log_10_ FDR > 1.3) are highlighted with enlarged data points, while a black outline marks the lead SNP within each locus.

Among the shared SNPs, we identified a total of 100 independent loci jointly associated with ASD and one of cardiometabolic phenotypes (conjFDR < 0.05), with some loci identified for several phenotypes, resulting in 68 unique independent loci (Fig. 2, Table S5). Independent genetic loci shared between ASD and metabolic traits were: 21 loci with BMI, 8 with T2D, 3 with TC, 5 with HDL, and for cardiovascular traits: 25 with SBP, 23 with DBP, 14 with PP, and 1 with CAD (Table 1). Notably, 20 of these loci were associated with ASD and more than one cardiometabolic phenotype, suggesting overlapping genetic risk factors across these conditions. Of the shared loci at conjFDR<0.05, 6 were novel for ASD (see Table S5).

We analyzed the effect directionality of the lead SNPs within the shared loci and found that 40% showed concordant effect directions between ASD and all studied traits, whereas 60% were discordant. Specifically, almost null and concordant effect directions were observed for metabolic traits: 48% of SNPs shared between ASD and BMI, 60% between ASD and HDL, 50% between ASD and T2D, and 66% between ASD and TC; and majority of discordant directions for cardiovascular traits. Only 48% between ASD and DBP, 36% between ASD and SBP, and 7% of the SNPs shared between ASD and PP showed concordant effects. The single SNP shared between ASD and CAD made it difficult to analyze effect percentage, but this was discordant effect direction (Table 1 and Table S5). These mixed effect directions were generally consistent with positive genetic correlation between metabolic traits, and negative genetic correlations with cardiovascular traits within the shared component.

### Functional analyses

Functional annotation of candidate SNPs shared between ASD and cardiometabolic phenotypes (conjFDR < 0.05) revealed that the majority were intronic or intergenic variants (Figure S3). Using FUMA positional mapping, these SNPs were mapped to 124 genes. Although no Gene Ontology (GO) terms were significantly enriched among these genes, GWAS catalog terms most frequently associated with genes shared between ASD and cardiometabolic phenotypes pertained to brain-related phenotypes, including neuroticism (FDR-P = 1.85e-17), Brain morphology (FDR-P = 4.04e-09), and General risk tolerance (FDR-P = 2.13e-07). Additionally, terms related to other phenotypes, such as meat consumption (FDR-P = 8.27e-22), Fish- and plant-related diet (FDR-P = 1.65e-12), and waist circumference adjusted for BMI (FDR-P = 9.84e-09), were also identified (Table S6). For cardiovascular traits, most enriched GWAS catalog terms were brain morphology (FDR-P = 1.86e-35) and neuroticism (FDR-P = 2.84e-23), among others. Also, some non-brain phenotypes were enriched, such as ulcerative colitis (FDR-P = 2.67e-16), and Crohn’s disease (FDR-P = 2.43e-10), among others (Table S7).

Furthermore, several SNPs exhibited potential functional consequences based on CADD scores. Among the lead SNPs, rs17814604 on chromosome 2 was a non-coding RNA intronic variant with the highest CADD score of 26.5, identified in the ASD-HDL pair. Additionally, rs325485 on chromosome 2, another non-coding RNA intronic variant, had a CADD score of 18.49 and was associated with both ASD and T2D. The lead SNP rs4841432 on chromosome 8 was an exonic variant of the *SOX7* gene with a CADD score of 17.85, associated with DBP and SBP. Furthermore, rs97668 was an intergenic variant with a CADD score of 13.12, associated with ASD and BMI. Overall, 86 candidate SNPs (conjFDR < 0.05) had CADD scores exceeding 12.37, including metabolic traits: 10 SNPs shared with BMI, 2 with HDL, 7 with T2D, and 2 with TC and cardiovascular traits: 1 with CAD, 75 with DBP, 60 with PP, 75 with SBP, (Table S8). Notably, the candidate SNP rs17651549 on chromosome 17, associated with PP, DBP, and SBP, was an exonic variant of the *MAPT* gene with the highest CADD score of 26.8.

Finally, using GREP, three significant (P < 0.05) enrichment groups of drugs were identified for genes shared by ASD and cardiometabolic phenotypes, such as the drugs for functional gastrointestinal disorders (A03; odds ratio = 14.6, P  =  0.01), antiepileptics (N03; OR = 9.5, P = 0.03), and antibacterials for systemic use (J01; OR = 27.9 P = 0.04, Table S9). For genes shared by ASD and cardiometabolic phenotypes, we found 13 unique genes that interacted with 104 drugs (Table S10). Notably, *MAPT* showed the greatest number of unique drugs (n  =  45), followed by *HDAC9* (n  =  13), *BLK* (n  =  12), and *CACNB2* (n  =  9).

## Discussion

This study provides new evidence for shared genetic overlap between ASD and metabolic and cardiovascular traits, advancing our understanding of the genetic landscape linking neurodevelopmental and cardiometabolic health. Using advanced genomic tools and large-scale GWAS datasets, we revealed overlaps in the genetic architecture of ASD and several cardiometabolic conditions, with mainly concordant effect directions between ASD and metabolic phenotypes, and discordant effect directions in cardiovascular traits related to blood pressure. We identified 100 shared loci and substantial genetic correlation of the overlapping variants, despite the negligible global genetic correlations observed between the traits. Interestingly, there was a clear pattern of concordant SNP effect directions between ASD and metabolic traits (BMI, T2D, TC, and HDL), while a discordant effect direction between ASD and cardiovascular traits involving blood pressure and pulse. These results provide valuable insights into potential shared molecular mechanisms underlying ASD and cardiometabolic and cardiovascular conditions.

Our findings revealed a polygenic overlap between ASD and metabolic traits, particularly BMI, highlighting a complex genetic relationship that goes beyond simple genetic correlation, and suggests a balanced mixture of concordant and discordant directions of effect among the shared variants, reflecting the apparent discrepancy observed between MiXeR and LDSC estimates [37]. This high degree of shared polygenicity may reflect overlapping biological pathways involved in both neurodevelopment and metabolic regulation, consistent with accumulating evidence of genetic links between neurodevelopmental disorders and BMI [27, 43, 44]. In the pleioFDR analyses, most overlapping loci also exhibited concordant directions of effect, suggesting a shared genetic predisposition for both ASD and metabolic dysfunction. This shared architecture is further underscored by concordant associations with lipid traits, including TC and HDL. Dysregulation of lipid metabolism is well-documented in ASD, with studies reporting elevated TC, low-density lipoprotein (LDL), and triglycerides in affected individuals [45]. Such disruptions in lipid homeostasis are critical given the involvement of cholesterol in brain function and development [46, 47]. Abnormal cholesterol and fatty acid metabolism in ASD has also been linked to oxidative stress and mitochondrial dysfunction, which may exacerbate neurodevelopmental deficits [48, 49]. These findings complement the shared genetic architecture identified for ASD and T2D, and suggest a role for metabolic processes in shaping neurodevelopmental outcomes, possibly mediated by pathways affecting insulin signaling, as previously suggested [50–52].

Our study also revealed significant genetic overlap between ASD and cardiovascular phenotypes involving blood pressure and pulse (SBP, DBP, PP) and CAD. Notably, this overlap was characterized by discordant directions of effect, with ASD-associated loci linked to lower blood pressure and pulse, while non-conclusive for CAD risk. This finding contrasts with the concordant genetic overlap observed between ASD and metabolic traits, suggesting distinct and potentially opposing biological pathways influencing metabolic and vascular health in ASD. In fact, the genetic determinants of blood pressure regulation are distinct from those influencing lipid metabolism and inflammation-driven CAD [53]. CAD is primarily a metabolic and inflammatory condition driven by atherosclerosis [20, 21], whereas blood pressure traits are influenced by vascular resistance, kidney function, and neurohormonal regulation [22, 54]. While our findings show a genetic overlap between ASD and blood pressure-related traits, suggesting shared mechanisms related to autonomic dysfunction [55, 56], the limited overlap with CAD (based on one locus) indicates that classic atherosclerotic pathways are not strongly implicated in ASD. These findings diverge from patterns observed in other psychiatric disorders, such as schizophrenia, which shows neutral or positive genetic correlations with both metabolic and cardiovascular traits [27]. This complexity may help to explain the heterogeneity in health outcomes observed in individuals with ASD [57]. While the average metabolic and cardiovascular risk in ASD is higher than in the general population [2, 17, 19], there is considerable individual variation. Our findings suggest that increased metabolic comorbidities in ASD may be primarily driven by genetic susceptibility, while blood-pressure related comorbidities appear to mainly reflect the contribution from environmental factors, lifestyle, and clinical characteristics.

Pleiotropic loci identified were mapped to 124 genes, with several lead SNPs exhibiting high CADD scores (CADD > 12.37), indicating deleterious effects. Some of these SNPs mapped to genes previously implicated in both neurodevelopmental and metabolic processes. For example, *SOX7* and *MAPT* (an exonic variant), which have been implicated in ASD and cardiovascular development [58–61]. Nonetheless, fine-mapping and functional interpretation within certain loci (such as 17q21.31) are inherently limited due to their complex LD structure. The enrichment of functional analyses for GWAS genes associated with neuroticism and brain morphology further highlights the involvement of shared pathways in ASD and brain development. Additionally, enriched pathways related to inflammatory and immune responses and metabolic processes, such as waist circumference, provide compelling evidence for the role of immune and metabolic dysregulation in the etiology of ASD and its comorbidities [62]. Our results support the growing body of literature on genetic pleiotropy as a driver of comorbidity patterns between ASD and metabolic and cardiovascular disorders [17] and parallel insights into the complex interplay between psychiatric conditions and cardiometabolic risk [63–65]. Despite being preliminary, our findings on drug repurposing also revealed nine genes linked to medications of potential therapeutic interest, including those used for functional gastrointestinal disorders and antiepileptic drugs. These exploratory results illustrate how cross-phenotype genetic analyses can reveal biological targets that may inform future therapeutic approaches for individuals with ASD and co-occurring cardiometabolic conditions.

Our study has certain limitations. First, although our findings support shared genetic architecture between ASD and cardiometabolic traits, some associations may reflect phenotypic mediation rather than true horizontal pleiotropy. Second, the power of ASD GWAS remains modest for MiXeR analysis, reflected in positive yet small AIC values. Third, MiXeR assumes comparable LD structure across GWAS datasets; discrepancies between sample LD and the reference panel may bias parameter estimates and prevent trans-ancestry analyses. Fourth, although MiXeR estimates of polygenicity and polygenic overlap do not depend on the sample size [34], the number of specific pleiotropic loci identified with pleioFDR is power-dependent and may vary for the same analyzed phenotypes depending of the GWAS sample sizes. Fifth, methods used to detect pleiotropy are limited by uncertainties in translation genetic loci into causal variants, which hinders the biological interpretation of the shared genetic variants [38]. Sixth, although both ASD and cardiometabolic traits show sex differences in prevalence and, in some cases, heritability [66], sex-stratified GWAS summary statistics were unavailable, preventing evaluation of sex-dependent effects. Finally, ASD is a highly heterogeneous condition with contributions from rare, highly penetrant variants and a polygenic background of common variation. While GWAS approaches are powerful for detecting shared polygenic architecture, they average across etiologically diverse subgroups. The common SNPs identified in this study only account for a small fraction of ASD liability. This emphasizes the importance of incorporating rare variants, environmental influences, and gene-environment interactions in future studies.

In conclusion, our findings enhance our understanding of comorbidity patterns in ASD and its shared genetic architecture with cardiometabolic traits. Specially, our results emphasize the importance of distinguishing metabolic -which exhibit shared polygenic risk with concordant effect directions- and blood pressure traits -which show polygenic overlap with discordant effect directions- when studying their association with ASD. These findings contribute to understanding the biological underpinnings of these co-occurring conditions and may serve as a basis for future targeted interventions aimed at reducing cardiometabolic complications in patients with ASD who are genetically at risk.

## Supplementary information

Supplementary Figures

Supplementary Tables

## Data Availability

Data supporting the findings of this study are openly available from an online repository or are available on request from the study authors. Please refer to Supplementary Material for further details.

## Code availability

All codes are freely available at https://github.com/precimed, https://github.com/precimed/mixer, and https://github.com/bulik/ldsc. Analyses were conducted in Python v3.5, Matlab R2020b and R Software v4.1. Functional annotation, and gene-set analysis were performed using FUMA (https://fuma.ctglab.nl/).
