# Supplementary material for: Genetic overlap and shared risk loci between autism spectrum disorder and cardiometabolic traits
Source: Mol Psychiatry. 2026 Apr 20;31(9):4874–81. doi: 10.1038/s41380-026-03563-x (PMC13441904; doi:10.1038/s41380-026-03563-x)
Supplement: Supplementary file 1 [file 41380_2026_3563_MOESM1_ESM.pdf]

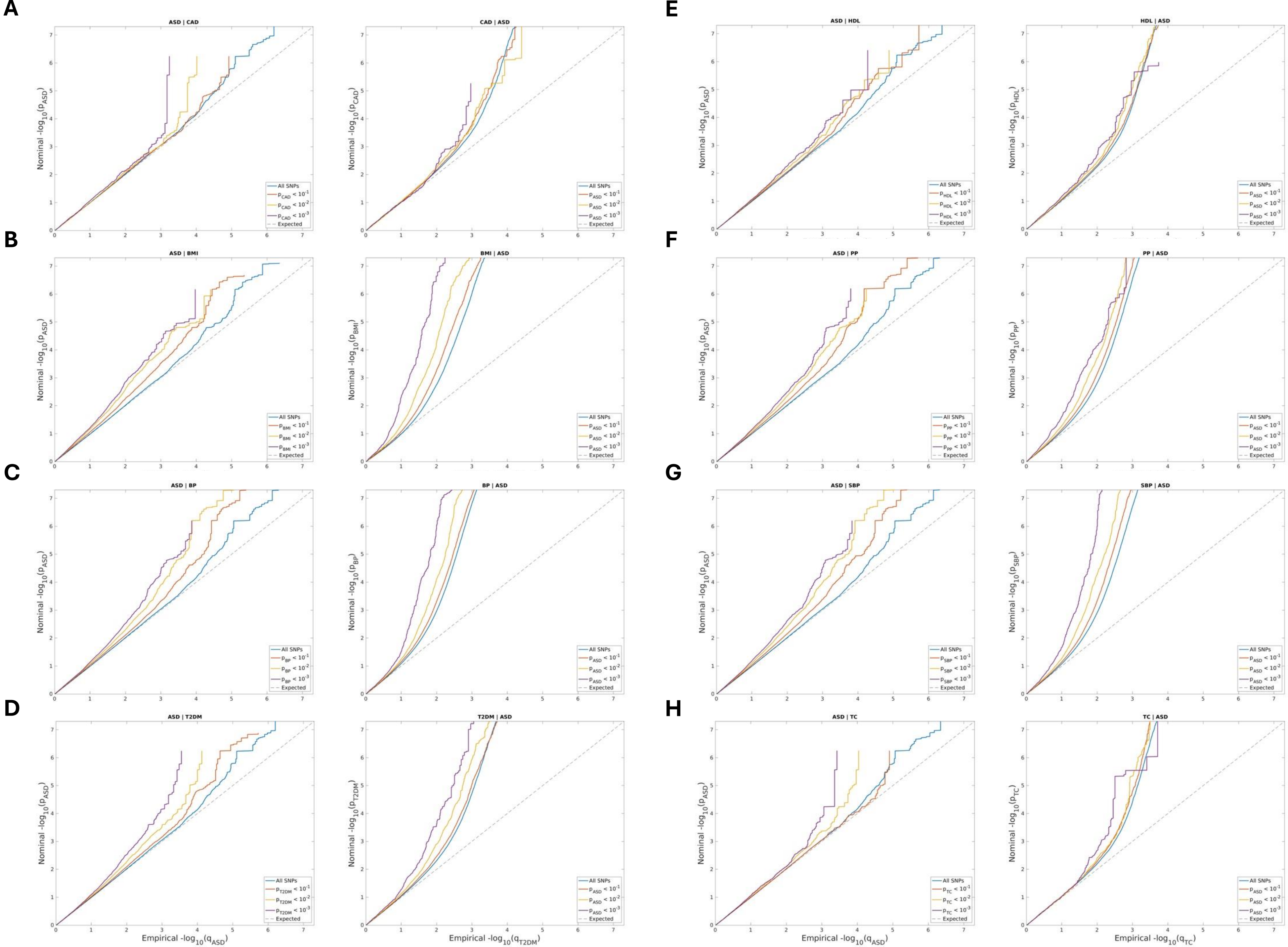

Figure S1. Conditional Q–Q plots illustrating cross-trait enrichment between ASD and cardiometabolic traits. The plots display the relationship between the expected and observed $-\log_{10}$ p-values for single-nucleotide polymorphisms (SNPs) associated with the primary phenotype (ASD) when the SNPs are stratified by their association with the conditional phenotype (cardiometabolic traits), and vice versa. There are four nested strata displayed: blue (all SNPs), orange (P conditional phenotype < 0.1), yellow (P conditional phenotype < 0.01), and purple (P conditional phenotype < 0.001). The dashed black line indicates the null distribution expected under no association. A progressive leftward deflection from this line for strata with increasing significance in the conditional phenotype reflects an enrichment of association signals, suggesting polygenic overlap between traits. The panels show ASD conditioned on: (A) CAD, (B) BMI, (C) BP, (D) T2DM, (E) HDL, (F) PP, (G) SBP, and (H) TC.

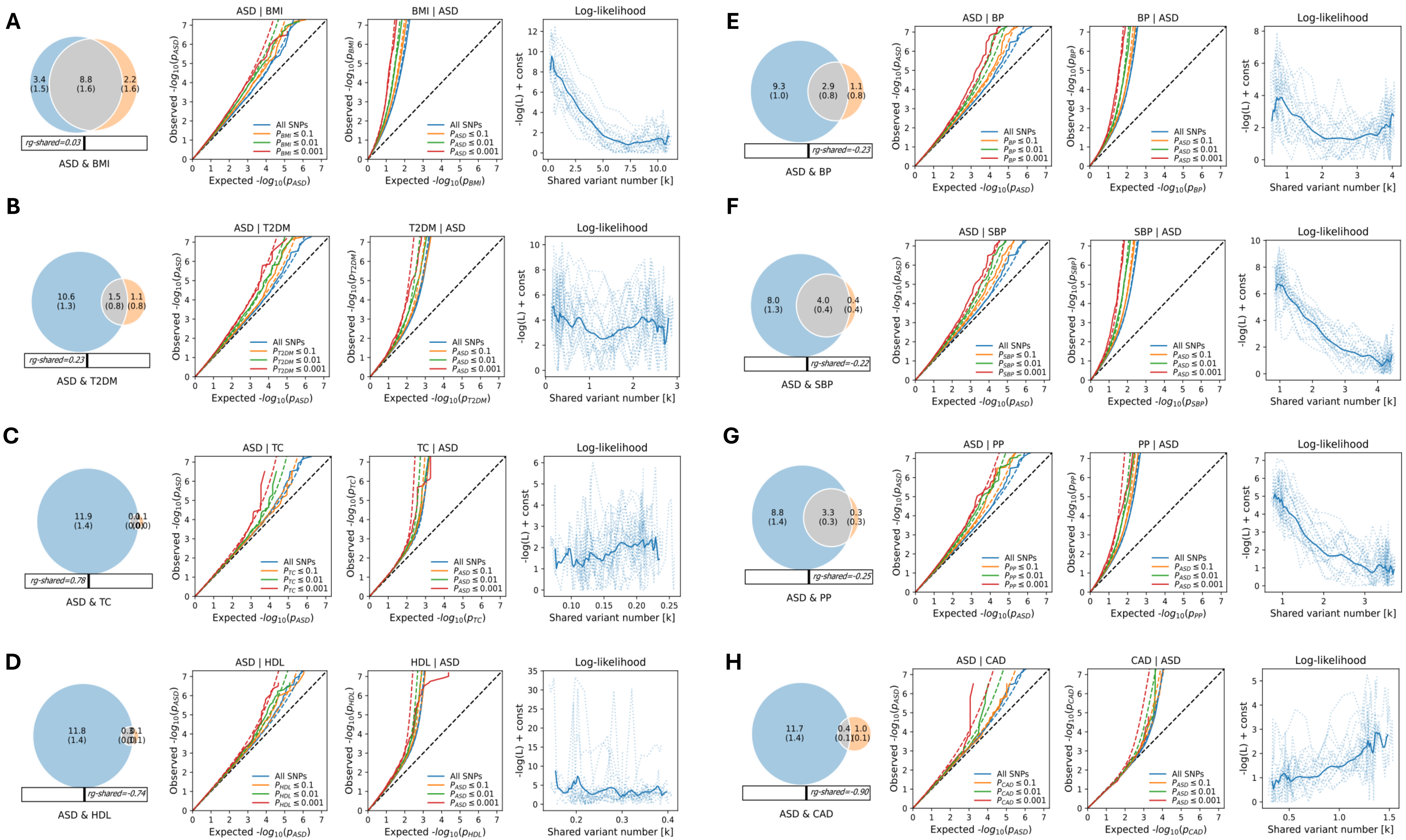

Figure S2. Polygenic overlap and cross-trait enrichment between ASD and cardiometabolic traits. Venn diagrams illustrate the estimated number of causal variants unique to ASD (blue), unique to cardiometabolic traits (orange), and shared between them (grey), as inferred by MiXeR. Numbers represent the estimated quantity of causal variants (in thousands), with standard errors shown in parentheses. Conditional Q–Q plots display the nominal versus empirical −log10 *p*-values (corrected for inflation) for ASD below the genome-wide significance threshold ($P < 5 \times 10^{-8}$), conditional on increasing levels of association with cardiometabolic traits ($P < 0.1$, $P < 0.01$, $P < 0.001$), and vice versa. The blue lines represent the distribution of all SNPs. Panels correspond to ASD and the following cardiometabolic traits: (A) BMI, (B) T2DM, (C) TC, (D) HDL, (E) BP, (F) SBP, (G) PP, and (H) CAD. rg-shared indicates the genetic correlation within the shared polygenic component.

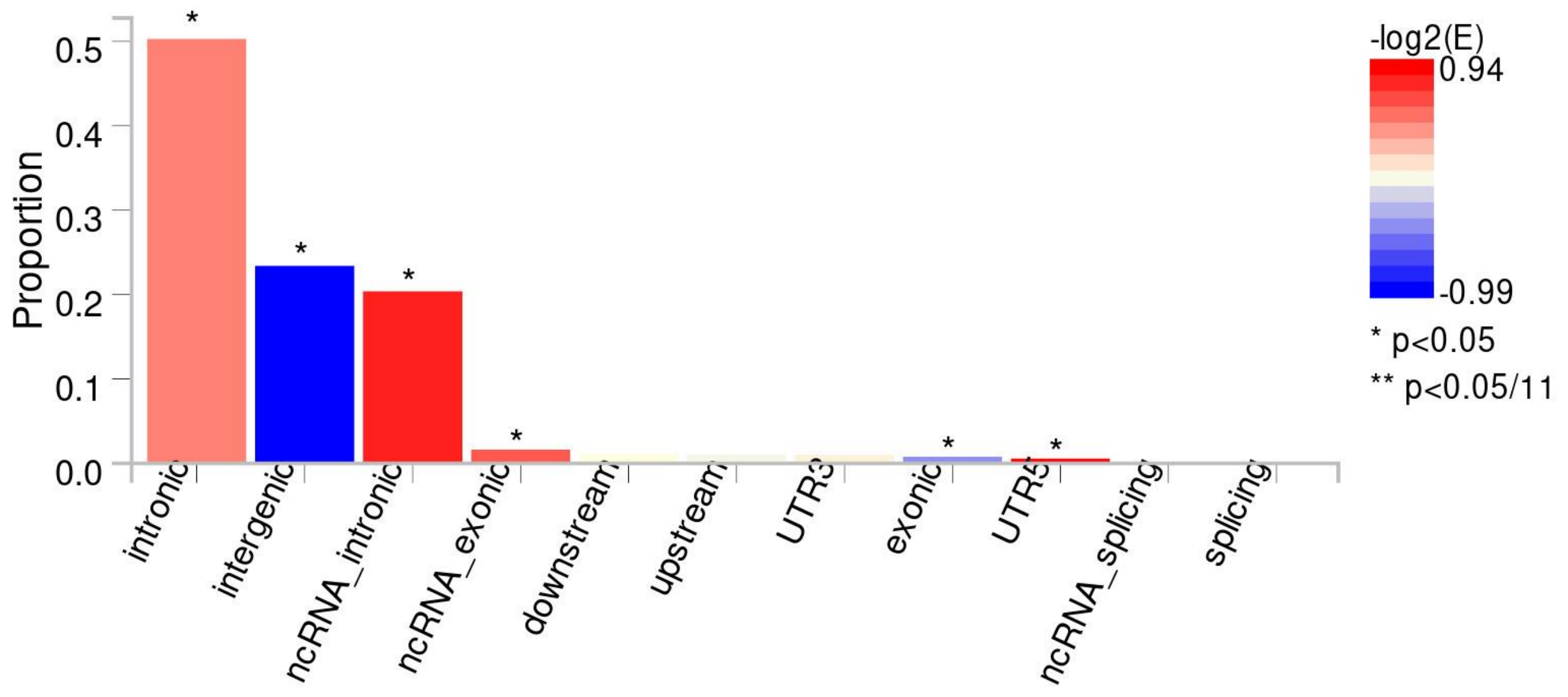

Figure S3. Functional annotation of SNPs associated with shared loci. The histogram shows the proportion of SNPs (including all SNPs in linkage disequilibrium with independent significant SNPs) assigned to each functional category by ANNOVAR. Bars are colored according to the $\log_2$ enrichment relative to all SNPs in the reference panel. Functional annotations and enrichment analyses were generated using FUMA.
